# Influence of the request and purchase of television advertised foods on dietary intake and obesity among children in China

**DOI:** 10.1186/s12889-021-11191-z

**Published:** 2021-06-12

**Authors:** Jinli Xian, Mao Zeng, Zhengjie Cai, Changxiao Xie, Yuqian Xie, Manoj Sharma, Yong Zhao, Zumin Shi

**Affiliations:** 1 School of Public Health and Management, Chongqing Medical University, Chongqing, 400016 China; 2 Research Center for Medicine and Social Development, Chongqing Medical University, Chongqing, 400016 China; 3 The Innovation Center for Social Risk Governance in Health, Chongqing Medical University, Chongqing, 400016 China; 4 The Second Clinical College, Chongqing Medical University, Chongqing, 400016 China; 5 Department of Environmental & Occupational Health, School of Public Health, University of Nevada, Las Vegas, NV 89119 USA; 6 Chongqing Key Laboratory of Child Nutrition and Health, Children’s Hospital of Chongqing Medical University, Chongqing, 400014 China; 7 Human Nutrition Department, College of Health Sciences, QU Health, Qatar University, Doha, Qatar

**Keywords:** Children, Television, Food advertisements, Purchasing behaviour, Dietary intake, Obesity

## Abstract

**Background:**

This study aims to examine the effects of the request and purchase of Television (TV) advertised foods on children’s dietary intake, overweight and obesity in China.

**Methods:**

Data from 1417 children (aged 6–17 years) in the 2011 China Health and Nutrition Survey were analysed. The request and purchase of TV advertised foods were assessed through the frequency of children’s requests to purchase TV advertised foods and the frequency of parents’ purchases of these advertised foods, as well as the frequency of children’s purchases of TV advertised foods. The height and weight of children were measured. Logistic regression models were used to identify the associations between the request and purchase of TV advertised foods and overweight/obesity of children.

**Results:**

The request and purchase of TV advertised foods were positively associated with children’s dietary intake of energy, protein, fat and carbohydrates. After adjusting for potential confounding factors, children’s request and purchase of TV advertised foods and parent’s purchase of TV advertised foods were positively associated with children’s overweight/obesity: odds ratio (OR) and 95% confidence interval (CI) for overweight/obesity were: 1.46 (1.01–2.11) for children purchasing advertised foods ≥1 time/week, 1.59 (1.15–2.18) for parents purchasing advertised foods for their children ≥1 time/week and 1.39 (1.00–1.95) for children requesting advertised foods ≥1 time/week.

**Conclusions:**

The request and purchase of TV advertised foods are associated with children’s dietary intake. Moreover, the request and purchase of TV advertised foods can increase the risk of overweight and obesity of children. Health education involving children’s request and purchase of TV advertised foods and parents’ purchase of TV advertised foods should be considered in China.

**Supplementary Information:**

The online version contains supplementary material available at 10.1186/s12889-021-11191-z.

## Background

Childhood obesity is likely to persist in adolescence and adulthood, leading to an increased risk of cardiovascular diseases, musculoskeletal disorders, cancers, insulin resistance and psychological effects [1]. Globally, over 340 million children and adolescents aged 5–19 years were overweight or obese in 2016 [1]. According to the Chinese National Survey on Students’ Constitution and Health, among students aged 7–18 years, the obesity prevalence increased from 0.1% in 1985 to 6.4% in 2014 [2]. The underlying cause of overweight and obesity is an energy imbalance between calorie intake and calorie expenditure [1].

Children are frequently exposed to Television (TV) food advertisements (ads). In a study of TV food ads targeting children in 11 countries, food ads comprised 11 to 29% of the total ads, and among the food ads, non-core foods (high in undesirable nutrients or energy, as defined by dietary standards) comprised 53 to 87% [3]. Powell et al. found that more than 95% of food and beverage TV ads seen on children’s programming were for products high in saturated fat, trans fat, sugar, and sodium [4]. This finding is similar to that of Rodd’s research [5].

TV food ads affected the food purchasing behaviours of children, which contributes greatly to childhood obesity. Previous studies have found that TV food ads increased children’s choose, requests and purchases for advertised foods [6–10]. Gbadamosi et al. found that TV ads were relevant to children’s consumption of goods and services, prompting children to request advertised products or brands, particularly advertised foods, from their parents in Africa [7]. This finding indicated that TV ads can influence children’s food preferences and purchase requests. In a study among children above 6 years old in Western countries, TV ads affected children’s requests for less nutritious products and their active participation in the decision-making process involved in purchases [10]. This may influence children’s overweight and obesity. Similarly, in China, a study of the relationship between TV watching and snacking behaviour among children and adolescents showed that watching TV, especially TV ads, was significantly related to the demand, purchase and consumption of snacks [9].

Studies have consistently shown that TV food ads affected children’s food purchase requests [7, 9, 10] and obesity [11–13]. However, little is known how the request and purchase of TV advertised foods affect children’s dietary intake and overweight and obesity. Thus, this study used the data from the 2011 China Health and Nutrition Survey (CHNS) to 1) explore the effect of the request and purchase of TV advertised foods on children’s dietary intake of energy, protein, fat and carbohydrates; 2) explore the effect of the request and purchase of TV advertised foods on overweight and obesity among children in China.

## Methods

### Study design

The CHNS is an ongoing open cohort, an international collaborative project between the Carolina Population Center at the University of North Carolina at Chapel Hill and the National Institute for Nutrition and Health at the Chinese Center for Disease Control and Prevention [14]. It was designed to examine the effects of the health, nutrition and family planning policies and programmes implemented by national and local governments and to see how the social and economic transformation of Chinese society affects the health and nutritional status of its population. The longitudinal CHNS has been conducted since 1989 in eight out of the 23 Chinese provinces (Guangxi, Guizhou, Henan, Hubei, Hunan, Jiangsu, Liaoning, Shandong), Heilongjiang province was enrolled as a ninth province in 1997 [15]. Three megacities (Beijing, Shanghai, Chongqing) have joined the study since 2011 [16]. The twelve participating provinces varied substantially in terms of geography, economic development, public resources, and health indicators, which makes the sample representative [14, 17]. A multistage, random cluster process was used to draw the sample in each of these provinces. Counties and cities in each province were stratified by income (low, middle and high), and a weighted sampling scheme was used to randomly select four counties and two cities in each province. Villages and townships within the counties and urban and suburban neighborhoods within the cities were selected randomly. In each community, 20 households were randomly selected and all household members were interviewed [15].

### Participants

Data from children aged 6–17 years who attended the 2011 CHNS were used in this study. In 2011, children aged 6–17 years were invited to answer the questions about the request and purchase of TV advertised foods. Children under the age of 10 could complete questionnaires (except dietary questionnaire) with the assistance of their parents [18]. A total of 1651 children aged 6–17 years were included in the study. Data were collected from participants during face-to-face interviews with trained interviewers at the participants’ home. We excluded those with missing information of height, weight, dietary intake of energy, fat, protein and carbohydrates (*n* = 133). Those had missing values of the following variables were also excluded: age, gender, residence, education, urbanisation index, per capita annual family income, ethnicity, the request and purchase of TV advertised foods (*n* = 101). The final sample included in the analysis was 1417 children.

### Measures

#### Exposure variables: the request and purchase of TV advertised foods

The request and purchase of TV advertised foods were assessed by the questions “Do you ask your parents to buy the kind of food you see on TV ads?”, “Do your parents purchase the advertised food for you?” and “Do you purchase the kind of food you see on TV ads by yourself?”. Respondents reported the frequency (“very seldom (<1 time/month)”, “seldom (1–3 times/month)”, “sometimes (1–2 times/week)”, “often (3–4 times/week)”, “very often (≥5 times/week)” and “unknown”) of these purchasing behaviours. Considering the reality of the question as well as the situation of the data itself, for each of the request and purchase of TV advertised foods, responses of “very seldom” and “seldom” were combined as “<1 time/week”; and responses of “sometimes”, “often” and “very often” were combined as “≥1 time/week”.

#### Outcome variables: dietary intake and overweight/obesity

Individual dietary data for the same three consecutive days were collected for all household members, regardless of age or relationship to the household head [16]. This was achieved by asking each individual, except children aged younger than 12, on a daily basis to report all food consumed at home and away from home on a 24-h recall basis [16]. If the children were not present, their caregivers would be required to contact them to obtain the children’s food consumption information. Using food models and picture aids, trained field interviewers recorded the type of food, amount, type of meal and place of consumption of all food items during the 24 h of the previous day [16]. For children younger than 12, the mother or a mother substitute who handled food preparation and feeding in the household was asked to recall the children’s food consumption. All field workers have been trained nutritionists who are otherwise professionally engaged in nutrition work in their own counties and who have participated in other national surveys [19]. Almost all interviewers have been graduates of post-secondary schools; many have had four-year degrees [19]. In addition, three days of specific training in the collection of dietary data have been provided for this survey [19].

Height and weight were measured in the 2011 CHNS survey. The height and weight of children were measured by at least two trained health workers who followed standard protocol and techniques, with one worker taking the measurements while a second health worker recording the readings [20]. Bodyweight was measured in light indoor clothing to the nearest tenth of a kilogram with a beam balance scale; height was measured without shoes to the nearest tenth of a centimeter, using a portable stadiometer [20]. The International Obesity Task Force cut-off of body mass index was used for defining overweight/obesity among children [21].

#### Covariates

Age, gender, ethnicity, education, residence, urbanisation index and per capita annual family income of the children were included in this study. Self-reported education as indicated in the questionnaire was allocated to one of three categories (illiterate/primary school, junior middle school, high middle school or higher) [22]. The urbanisation index was recoded into tertiles (low, medium, and high) [22]. Per capita annual family income was recoded into tertiles (low, medium, and high) [22].

### Data analyses

Descriptive statistics were used for the sample characteristics. The categorical variables were described using frequency and percentage, and the continuous variables were described using mean and standard deviation (SD). Mann-Whitney test was used to examine the relationships between age, dietary intake of energy, fat, protein, carbohydrates and overweight and obesity among children. Mann-Whitney test was also performed to examine the relationships between the request and purchase of TV advertised foods and dietary intake of energy, fat, protein and carbohydrates. To determine if the relationships between the request and purchase of TV advertised foods and overweight and obesity existed, odds ratio (OR) and 95% confidence interval (CI) for the outcome variable were calculated using binary logistic regression. Two multivariable models for each exposure variable were used: Model 1 was adjusted for age, gender, dietary intake of energy; Model 2 was further adjusted for ethnicity, education, income, urbanisation index and residence. The Pearson goodness-of-fit test indicated that all logistic regression models used in this study were good fits (*P* > 0.05). The variables adjusted in the multivariable models were either known risk factor of children’s overweight and obesity [1, 23] or sociodemographic factors. All statistical tests were conducted using STATA software (Version 12, StataCorp, College Station, TX, USA). Statistical significance was considered when *P* < 0.05 (two-sided).

## Results

### Sample characteristics of participants

Table 1 presents the sample characteristics of participants. The mean age of the sample was 10.9 (SD 3.3). Among participants, 63.1% lived in rural areas. The prevalence of overweight/obesity was 17.9%. Overall, 18.3% of the children reported they requested advertised foods from their parents ≥1 time/week, 16.9% purchased advertised foods by themselves ≥1 time/week and 21.1% reported that their parents purchased advertised foods for them ≥1 time/week.

**Table 1:** Sample characteristics of participants (*n* = 1417)

Variables		Overweight/Obesity		***P***-value
	Total populations	No (***n*** = 1164)	Yes (***n*** = 253)	
	n (%) or Mean ± SD	n (%) or Mean ± SD	n (%) or Mean ± SD	
**Age (year)**	10.9 ± 3.3	11.0 ± 3.3	10.2 ± 3.1	< 0.001
**Gender**				
Boy	725 (51.2%)	570 (49.0%)	155 (61.3%)	< 0.001
Girl	692 (48.8%)	594 (51.0%)	98 (38.7%)	
**Ethnicity**				
Han	1229 (86.7%)	995 (85.5%)	234 (92.5%)	0.003
Minority	188 (13.3%)	169 (14.5%)	19 (7.5%)	
**Education**				
Illiterate/primary school	895 (63.2%)	711 (61.1%)	184 (72.7%)	0.002
Junior middle school	368 (26.0%)	316 (27.1%)	52 (20.6%)	
High middle school or higher	154 (10.9%)	137 (11.8%)	17 (6.7%)	
**Urbanisation index**				
Low	473 (33.4%)	410 (35.2%)	63 (24.9%)	0.002
Medium	474 (33.5%)	388 (33.3%)	86 (34.0%)	
High	470 (33.2%)	366 (31.4%)	104 (41.1%)	
**Urban or rural**				
Urban	523 (36.9%)	414 (35.6%)	109 (43.1%)	0.025
Rural	894 (63.1%)	750 (64.4%)	144 (56.9%)	
**Per capita annual family income**				
Low	473 (33.4%)	402 (34.5%)	71 (28.1%)	< 0.001
Medium	472 (33.3%)	400 (34.4%)	72 (28.5%)	
High	472 (33.3%)	362 (31.1%)	110 (43.5%)	
**Children requested advertised foods from parents** (time/week)				
< 1	1157 (81.7%)	968 (83.2%)	189 (74.7%)	0.002
≥ 1	260 (18.3%)	196 (16.8%)	64 (25.3%)	
**Parents purchased advertised foods for their children** (time/week)				
< 1	1118 (78.9%)	940 (80.8%)	178 (70.4%)	< 0.001
≥ 1	299 (21.1%)	224 (19.2%)	75 (29.6%)	
**Children purchased advertised foods** (time/week)				
< 1	1177 (83.1%)	975 (83.8%)	202 (79.8%)	0.132
≥ 1	240 (16.9%)	189 (16.2%)	51 (20.2%)	
**Energy intake** (kcal/d)	1554.2 ± 569.1	1534.3 ± 567.2	1645.6 ± 570.2	0.004
**Fat intake** (g/d)	58.3 ± 31.3	57.1 ± 31.2	63.7 ± 31.0	< 0.001
**Protein intake** (g/d)	53.9 ± 21.9	52.9 ± 21.7	58.5 ± 22.6	< 0.001
**Carbohydrates intake** (g/d)	203.2 ± 85.9	201.9 ± 85.2	209.3 ± 88.9	0.254

### Association of the request and purchase of TV advertised foods and dietary intake

Table 2 indicated that children whose parents purchased advertised foods for them ≥1 time/week had higher dietary intake of energy, protein and fat than children whose parents purchase advertised foods for them < 1 time/week (*P* < 0.05). Children who purchased advertised foods ≥1 time/week had higher dietary intake of energy, protein, fat and carbohydrates than children who purchase advertised foods < 1 time/week (*P* < 0.01). In addition, children who requested for advertised foods from their parents ≥1 time/week had higher dietary intake of protein (*P* < 0.05).

**Table 2:** Association of the request and purchase of TV advertised foods and dietary intake among children attending the CHNS (*n* = 1417)

Independent variables	N	Dietary intake			
		Energy intake (kcal/d), mean (SD)	Fat intake (g/d), mean (SD)	Protein intake (g/d), mean (SD)	Carbohydrates intake (g/d), mean (SD)
**Children requested advertised foods from their parents** (time/week)					
< 1	1157	1550.1 (571.9)	58.0 (31.5)	53.4 (22.0)	203.4 (86.8)
≥ 1	260	1572.5 (557.4)	59.7 (30.4)	56.1 (21.4)	202.4 (82.3)
***P-*value**		0.548	0.313	0.040	0.993
**Parents purchased advertised foods for their children** (time/week)					
< 1	1118	1534.8 (562.1)	57.5 (31.3)	52.6 (21.6)	201.4 (85.8)
≥ 1	299	1626.7 (590.1)	61.3 (30.9)	58.6 (22.7)	209.7 (86.1)
***P-*value**		0.022	0.025	< 0.001	0.124
**Children purchased advertised foods** (time/week)					
< 1	1177	1528.1 (557.1)	57.2 (31.1)	52.8 (21.4)	200.3 (84.6)
≥ 1	240	1682.4 (610.0)	63.7 (31.5)	59.3 (23.6)	217.5 (91.2)
***P*-value**		< 0.001	0.001	< 0.001	0.006

The age stratified analysis found that among children aged 15–17 years, the request and purchase of TV advertised foods were not significantly associated with children’s dietary intake of energy, protein, fat and carbohydrates (Supplement Table 1). The association between the request and purchase of TV advertised foods and children’s dietary intake of energy, protein, fat and carbohydrates among children aged 6–14 years were similar to that among children aged 6–17 years (Supplement Table 2).

### Logistic regression analyses of the association between the request and purchase of TV advertised foods and children’s overweight and obesity

Logistic regression analyses (Table 3) showed the relationship between the request and purchase of TV advertised foods and children’s overweight and obesity. When adjusting for age, gender and intake of energy, children who requested advertised foods ≥1 time/week (OR = 1.54; 95% CI 1.11–2.13) or purchased advertised foods ≥1 time/week (OR = 1.50; 95% CI 1.05–2.16) were more likely to become overweight and obese than children who requested advertised foods < 1 time/week or purchased advertised foods < 1 time/week; and children whose parents purchased advertised foods for them ≥1 time/week had a higher risk of overweight and obesity (OR = 1.70; 95% CI 1.25–2.32) than children whose parents purchase advertised foods for them < 1 time/week. After further adjusting for ethnicity, education, income, urbanisation index and residence, the risk of overweight and obesity increased to 1.46 times for children who purchased advertised foods ≥1 time/week compared to those purchased advertised foods < 1 time/week (OR = 1.46; 95% CI 1.01–2.11); and the risk of overweight and obesity increased to 1.59 times for children whose parents purchased advertised foods for them ≥1 time/week compared to those whose parents purchased advertised foods for them < 1 time/week (OR = 1.59; 95% CI 1.15–2.18). However, in this model, children who requested advertised foods from their parents ≥1 time/week were not significantly related to the risk of childhood overweight and obesity (*P* > 0.05).

**Table 3:** Association (OR, 95%CI) between the request and purchase of TV advertised foods and overweight and obesity among children attending the CHNS (*n* = 1417)

Independent variables	Model 1^a^	Model 2^b^
	OR (95% CI)	OR (95% CI)
**Children requested advertised foods from their parents** (time/week)		
< 1	Reference	Reference
≥ 1	1.54 (1.11–2.13)*	1.39 (1.00–1.95)
**Parents purchased advertised foods for their children** (time/week)		
< 1	Reference	Reference
≥ 1	1.70 (1.25–2.32)**	1.59 (1.15–2.18)**
**Children purchased advertised foods** (time/week)		
< 1	Reference	Reference
≥ 1	1.50 (1.05–2.16)*	1.46 (1.01–2.11)*

^a^ Model 1: adjusted for age, gender, intake of energy

^b^ Model 2: additional adjustment for ethnicity, education, income, urbanisation index, residence (urban/rural)

A total of 6 logistic regression models were used, two models for each exposure variable.

* P < 0.05, ** P < 0.01

The age stratified analysis found that among children aged 15–17 years, the request and purchase of Television advertised foods were not significantly associated with overweight and obesity (Supplement Table 3). However, among children aged 6–14 years, the request and purchase of Television advertised foods were positively associated with children’s overweight and obesity (Supplement Table 4).

## Discussion

Approximately 20% of the children requested advertised foods from their parents, purchased advertised foods, and reported that their parents purchased advertised foods for them. One important reason may be that the marketing strategies of repetition and enticement in TV food ads have the potential to influence the purchase intentions of children and parents [24]. Marketing strategies in TV food ads, such as using celebrity endorsers or cartoon characters to promote products, designing snack packages with cartoon images or adding small toys in snacks, may have a promotion effect on the food purchasing behaviours of children and their parents [25]. Previous studies have consistently demonstrated that TV food ads can increase children’s requests and purchases of advertised food [6–10]. And Television food advertising was considered as a powerful medium predisposing the mind of children to non-core foods (foods high in fat, refined sugars, and salt) through appealing TV commercials, promoting purchase requests and generating unhealthy food preferences in early childhood [26, 27]. In the present study, 21.1% of the children reported that their parents purchased advertised foods for them. Previous studies have found that parents’ purchasing behaviour could be affected by children’ purchasing requests [28, 29]. Some studies have shown that parents usually have negative attitudes towards ads; they believe that unhealthy foods in ads may have harmful influences on children’s dietary habits [30–32]. However, other studies found that parents’ behaviour of purchasing snacks for their children was greatly influenced by media ads and lacked rationality, which may be due to parents’ lack of knowledge of the nutritional information in children’s snacks [32, 33]. In addition, a previous study demonstrated that parents’ positive discussions with their children about ads can increase children’s understanding of ads and offset the negative effects of ads to a certain degree [34]. Hence, strengthening the health education on TV food ads for parents and children, restricting children’s purchasing behaviours of unhealthy advertised foods and training children to distinguish between healthy and unhealthy food ads would help children develop correct cognition of advertised foods. In addition, parents correctly interpret the content of TV food ads for their children may also be an effective measure. In fact, many developed countries have restricted TV food ads targeted to children [35]. However, it is not the case in China. The ‘Advertising Law of the People’s Republic of China’ stipulates that the content of ads for children under the age of 14 should not ‘persuade children to ask their parents to buy advertising goods or services’. [36] The Chinese government should formulate relevant policies to restrict unhealthy food ads to be broadcast on various media platforms, specifically on children’s programmes. Some public service ads or tips related to healthy dietary habits (i.e. consuming less high-sodium, high-sugar or high-fat foods) should be inserted in children’s programmes.

This study demonstrated that the request and purchase of TV advertised foods ≥1 time/week were positively associated with children’s dietary intake of energy, protein, fat and carbohydrates. Advertising foods of poor nutritional quality can influence children’s food preferences and make children tend to choose food rich in fat, refined sugar and sodium [26]. This inclination may result in higher dietary energy and fat intake in children who requested or purchased advertised foods ≥1 time/week than children who requested or purchased advertised foods < 1 time/week in this study. Moreover, the amount of time children spend watching TV was associated with the number of their requests for advertised foods, and the likelihood that parents would purchase these foods [6, 8]. This finding may be a reason for a previous conclusion that longer TV viewing time was associated with a higher intake of energy and fat among children [6]. Simultaneously, the current study found that children who purchased advertised foods had higher protein intake, which may be due to the ads of milk and dairy products targeted to children. Milk is rich in a variety of nutrients, especially protein, calcium and vitamins, and its protein digestibility rate is high (92–98%), which can increase children’s protein intake and contribute to the development and growth of children [37]. A previous study found that the popularity of dairy product ads in various media had a positive impact on people’s understanding of the benefits of consuming dairy products and facilitated milk purchase decisions [38]. This finding implies that the government should implement policies to promote healthy food ads on various media platforms, thereby increasing the recognition and consumption of healthy foods among children. However, a recent study found that individual food purchasing was an inaccurate basis for characterising dietary intake of energy and nutrients, which may be influenced by food wastage, non-consumption of purchased foods and consuming foods that were purchased by others [39]. Even if an individual consumes only self-purchased foods, it is difficult to fully calculate the nutritional content of the individual consumes by using individual food purchases, because each dietary recall represents only a “sample” from all purchased foods, not the nutritional content of the entire purchased set [39]. Therefore, the request and purchase of TV advertised foods could only reflect the dietary intake of children on a limited level.

The request and purchase of TV advertised foods ≥1 time/week were positively associated with children’s overweight and obesity, which may be a reason for the positive association between screen time and obesity [6, 40, 41]. This finding may have several possible explanations. Firstly, previous studies have shown that children were exposed to a considerable number of TV food ads, predominantly of foods high in fat, sodium and sugar [4, 42]. Purchasing and consuming these foods may be a reason for the increased risk of obesity in children [43]. These findings are consistent with the result of this study that overweight or obese children had a higher proportion of purchasing advertised foods than children who are not overweight or obese. Secondly, the present study showed that the request and purchase of TV advertised foods ≥1 time/week resulted in higher dietary intake of energy, protein and fat. According to previous studies, excessive dietary energy intake is a major factor in the development of obesity in children [44]; and high fat intake appears to be causally related to overeating and obesity, at least in susceptible individuals [45]. Thirdly, in developing countries such as China, older primary caregivers, such as grandparents, appear to be susceptible and receptive to children’s food purchasing requests [46]. Thus, these primary caregivers are likely to give in to children’s purchasing requests for unhealthy advertised foods, which may influence children’s eating habits and increase the risk of childhood obesity.

The age stratified analysis found that among the children aged 15–17 years, the request and purchase of TV advertised foods were not significantly associated with children’s dietary intake and overweight/obesity. This result may be influenced by the relatively small sample size of children aged 15–17 years. However, among the children aged 6–14 years, the request and purchase of TV advertised foods were positively associated with children’s dietary intake and overweight/obesity. The reason for this result may be that advertising is aimed at specific targets and the same ad may have a different impact on children in different age stages. Younger children may be more easily affected by TV food ads as their awareness of advertised foods may be insufficient.

Several limitations of this study need to be mentioned. Firstly, the cross-sectional data analysed in the study restricts conclusions about a causal relationship between the request and purchase of TV advertised foods and dietary intake and overweight and obesity among children. Secondly, the detailed information about the advertised products (characteristics, kind of food, brand, etc) was not collected. Thus, the use of “TV food advertisements” in this study is nonspecific and may reduce the consistency of the research. In a future study, we hope that information about the advertised foods can be obtained to establish the relationship between the food advertisement itself, the purchase, and the consumption of the advertised foods and obesity among children. Thirdly, the request and purchase of TV advertised foods were self-reported by children. Hence, responses could be influenced by acquiescence bias, extreme response bias or dishonesty. Therefore, the result of the study should be approached with caution.

## Implications for research and practice

This analysis indicated that the request and purchase of TV advertised foods have a positive effect on children’s dietary intake of energy, protein and fat. Moreover, the request and purchase of TV advertised foods are positively associated with children’s overweight and obesity. The findings stress the importance of targeting children aged 6–17 years, particularly children aged 6–14 and their parents to promote rational dietary intake and reduce the risk of obesity in children via reducing requesting and purchasing unhealthy TV advertised foods. Future research would benefit from longitudinal studies with more precise assessments of purchasing and consuming what kinds of foods seen on TV food ads to further understand the influences that contribute to the development of children’s dietary intake and childhood obesity. Finally, future obesity prevention or dietary intake interventions may consider focusing on healthy TV food ads as an innovative way to promote and target healthy eating and food purchasing behaviours. The result of the study would reflect that Chinese government should formulate regulations on TV food ads; health educators should conduct TV food ads related education or interventions to promote healthy food purchasing behaviours and dietary intake and reduce the risk of childhood obesity.

## Conclusion

This analysis constitutes the first large-scale study of the relationship between the request and purchase of TV advertised foods and dietary intake and overweight/ obesity among children in China. The results indicated that the request and purchase of TV advertised foods have a positive effect on dietary intake of energy, protein, fat and carbohydrates of children. Moreover, the request and purchase of TV advertised foods are positively associated with children’s overweight and obesity. Hence, conducting studies in China that can track more nutritional behaviours related to TV food ads is necessary.

## Supplementary Information

**Additional file 1 Supplement Table 1.** Association of the request and purchase of TV advertised foods and dietary intake among children aged 15–17 attending the CHNS (*n* = 241). **Supplement Table 2.** Association of the request and purchase of TV advertised foods and dietary intake among children aged 6–14 attending the CHNS (*n* = 1176). **Supplement Table 3.** Association (OR, 95%CI) between the request and purchase of TV advertised foods and overweight and obesity among children aged 15–17 attending the CHNS (n = 241). **Supplement Table 4.** Association (OR, 95%CI) between the request and purchase of TV advertised foods and overweight and obesity among children aged 6–14 attending the CHNS (n = 1176).

## Data Availability

The datasets analysed during the current study are available in the CHNS official website: https://www.cpc.unc.edu/projects/china/data/datasets. And public access to the database is open.

## Abbreviations

TV: Television
OR: Odds ratio
CI: Confidence interval
ads: Advertisements
CHNS: China Health and Nutrition Survey
SD: Standard deviation
