# Supplementary material for: Influence of the request and purchase of television advertised foods on dietary intake and obesity among children in China
Source: BMC Public Health. 2021 Jun 12;21:1130. doi: 10.1186/s12889-021-11191-z (PMC8199678; doi:10.1186/s12889-021-11191-z)
Supplement: Supplementary file 1 — Additional file 1 Supplement Table 1. Association of the request and purchase of TV advertised foods and dietary intake among children aged 15–17 attending the CHNS (n = 241). Supplement Table 2. Association of the request and purchase of TV advertised foods and dietary intake among children aged 6–14 attending the CHNS (n = 1176). Supplement Table 3. Association (OR, 95%CI) between the request and purchase of TV advertised foods and overweight and obesity among children aged 15–17 attending the CHNS (n = 241). Supplement Table 4. Association (OR, 95%CI) between the request and purchase of TV advertised foods and overweight and obesity among children aged 6–14 attending the CHNS (n = 1176). [file 12889_2021_11191_MOESM1_ESM.docx]

**Supplement table 1.** Association of the request and purchase of TV advertised foods and dietary intake among children aged 15–17 attending the CHNS (n = 241).

|  |  | **Dietary intake** | | | |
| --- | --- | --- | --- | --- | --- |
| **Independent variables** | **N** | **Energy intake (kcal/d), mean (SD)** | **Fat intake**  **(g/d), mean (SD)** | **Protein intake**  **(g/d), mean (SD)** | **Carbohydrates intake (g/d), mean (SD)** |
| **Children requested advertised foods from their parents** (time/week) |  |  |  |  |  |
| <1 | 202 | 1,797.5 (607.2) | 68.5 (34.4) | 63.2 (23.3) | 231.3 (90.9) |
| ≥1 | 39 | 1,798.8 (657.9) | 64.9 (39.4) | 61.8 (20.5) | 241.6 (99.5) |
| **P-value** |  | 0.765 | 0.275 | 0.785 | 0.802 |
| **Parents purchased advertised foods for their children** (time/week) |  |  |  |  |  |
| <1 | 187 | 1,791.6 (598.2) | 68.8 (34.8) | 62.4 (22.1) | 230.0 (89.9) |
| ≥1 | 54 | 1,818.7 (672.6) | 64.9 (37.0) | 64.7 (25.1) | 243.4 (100.0) |
| **P-value** |  | 0.949 | 0.321 | 0.746 | 0.510 |
| **Children purchased advertised foods** (time/week) |  |  |  |  |  |
| <1 | 167 | 1,786.0 (617.8) | 68.1 (36.5) | 62.3 (23.0) | 230.7 (90.2) |
| ≥1 | 74 | 1,824.2 (609.4) | 67.7 (32.4) | 64.4 (22.4) | 238.2 (97.0) |
| **P-value** |  | 0.601 | 0.833 | 0.493 | 0.698 |

**Supplement table 2.** Association of the request and purchase of TV advertised foods and dietary intake among children aged 6–14 attending the CHNS (n = 1176).

|  |  | **Dietary intake** | | | |
| --- | --- | --- | --- | --- | --- |
| **Independent variables** | **N** | **Energy intake (kcal/d), mean (SD)** | **Fat intake**  **(g/d), mean (SD)** | **Protein intake**  **(g/d), mean (SD)** | **Carbohydrates intake (g/d), mean (SD)** |
| **Children requested advertised foods from their parents** (time/week) |  |  |  |  |  |
| <1 | 955 | 1,497.7 (550.4) | 55.8 (30.4) | 51.3 (21.2) | 197.5 (84.7) |
| ≥1 | 221 | 1,532.6 (529.5) | 58.8 (28.5) | 55.1 (21.4) | 195.4 (77.1) |
| **P-value** |  | 0.287 | 0.071 | 0.010 | 0.976 |
| **Parents purchased advertised foods for their children** (time/week) |  |  |  |  |  |
| <1 | 931 | 1,483.2 (540.3) | 55.2 (30.1) | 50.6 (20.9) | 195.7 (83.9) |
| ≥1 | 245 | 1,584.4 (563.1) | 60.5 (29.4) | 57.3 (21.9) | 202.3 (81.1) |
| **P-value** |  | 0.013 | 0.004 | <0.001 | 0.181 |
| **Children purchased advertised foods** (time/week) |  |  |  |  |  |
| <1 | 1,010 | 1,485.4 (534.9) | 55.4 (29.8) | 51.2 (20.8) | 195.2 (82.6) |
| ≥1 | 166 | 1,619.2 (601.3) | 61.9 (31.1) | 57.0 (23.8) | 208.2 (87.2) |
| **P-value** |  | 0.006 | 0.006 | 0.005 | 0.057 |

**Supplement table 3.** Association (OR, 95%CI) between the request and purchase of TV advertised foods and overweight and obesity among children aged 15–17 attending the CHNS (n = 241).

|  | Model 1^a^ |  | Model 2^b^ |
| --- | --- | --- | --- |
| **Independent variables** | OR (95% CI) |  | OR (95% CI) |
| **Children requested advertised foods from their parents** (time/week) |  |  |  |
| <1 | Reference |  | Reference |
| ≥1 | 1.07 (0.37–3.09) |  | 1.16 (0.39–3.41) |
| **Parents purchased advertised foods for their children** (time/week) |  |  |  |
| <1 | Reference |  | Reference |
| ≥1 | 0.62 (0.22–1.73) |  | 0.59 (0.21–1.69) |
| **Children purchased advertised foods** (time/week) |  |  |  |
| <1 | Reference |  | Reference |
| ≥1 | 0.63 (0.25–1.55) |  | 0.64 (0.25–1.63) |

^a^ Model 1: adjusted for age, gender, intake of energy.

^b^ Model 2: additional adjustment for ethnicity, education, income, urbanisation index, residence (urban/rural).

A total of 6 logistic regression models were used, two models for each exposure variable.

* P < 0.05, ** P < 0.01.

**Supplement table 4.** Association (OR, 95%CI) between the request and purchase of TV advertised foods and overweight and obesity among children aged 6–14 attending the CHNS (n = 1176).

|  | Model 1^a^ |  | Model 2^b^ |
| --- | --- | --- | --- |
| **Independent variables** | OR (95% CI) |  | OR (95% CI) |
| **Children requested advertised foods from their parents** (time/week) |  |  |  |
| <1 | Reference |  | Reference |
| ≥1 | 1.61 (1.14–2.28)** |  | 1.44 (1.01–2.06)* |
| **Parents purchased advertised foods for their children** (time/week) |  |  |  |
| <1 | Reference |  | Reference |
| ≥1 | 1.95 (1.40–2.71)** |  | 1.82 (1.30–2.55)** |
| **Children purchased advertised foods** (time/week) |  |  |  |
| <1 | Reference |  | Reference |
| ≥1 | 1.83 (1.23–2.71)** |  | 1.78 (1.19–2.65)** |

^a^ Model 1: adjusted for age, gender, intake of energy.

^b^ Model 2: additional adjustment for ethnicity, education, income, urbanisation index, residence (urban/rural).

A total of 6 logistic regression models were used, two models for each exposure variable.

* P < 0.05, ** P < 0.01.
